# Alternative protein sources: science powered startups to fuel food innovation

**DOI:** 10.1038/s41467-024-47091-0

**Published:** 2024-05-28

**Authors:** Elena Lurie-Luke

**Affiliations:** Department of Biosciences, Durham University, DH1 3LE Durham, UK

**Keywords:** Agriculture, Business, Tissue engineering

## Abstract

Harnessing the potential of considerable food security efforts requires the ability to translate them into commercial applications. This is particularly true for alternative protein sources and startups being on the forefront of innovation represent the latest advancements in this field.

## Abstract (web summary)

Harnessing the potential of considerable food security efforts requires the ability to translate them into commercial applications. In this Perspective, the author explores the alternative protein source start-up landscape.

## Introduction

In 2022 almost 735 million people, 9.2% of the global population, were undernourished^1^ and as we are entering the second quarter of this century, the world faces a formidable task of feeding a growing population projected to reach 10 billion by 2050^2^. It is further aggravated by substantial food losses due to climate change and crop diseases^3^. To address this task requires a 50% increase in global food production in the next 25 years^2^ and the significance of food innovation becoming increasingly evident in order to create solutions tackling the urgency and scale of this food security challenge.

The World Resources Report ‘Creating a Sustainable Food Future’^4^ identified 22 solutions to address this need, which were grouped into five categories: (1) reduction of growth in demand for food and other agricultural products; (2) increase in food production without expanding agricultural land; (3) protection and restoration of natural ecosystems; (4) increase in fish supply; and (5) reduction of greenhouse gasses emissions from agricultural production. To implement these solutions, supporting the sustainable development goals^4^ and also addressing the food security challenge, require new technologies and product innovations, i.e., it is essential to find innovative ways to produce and distribute food more efficiently and sustainably. Achieving this goal can involve developing novel farming techniques (precision agriculture), exploring alternative protein sources, or implementing smart technologies to reduce waste and optimise food supply chains.

**Fig. 1:**
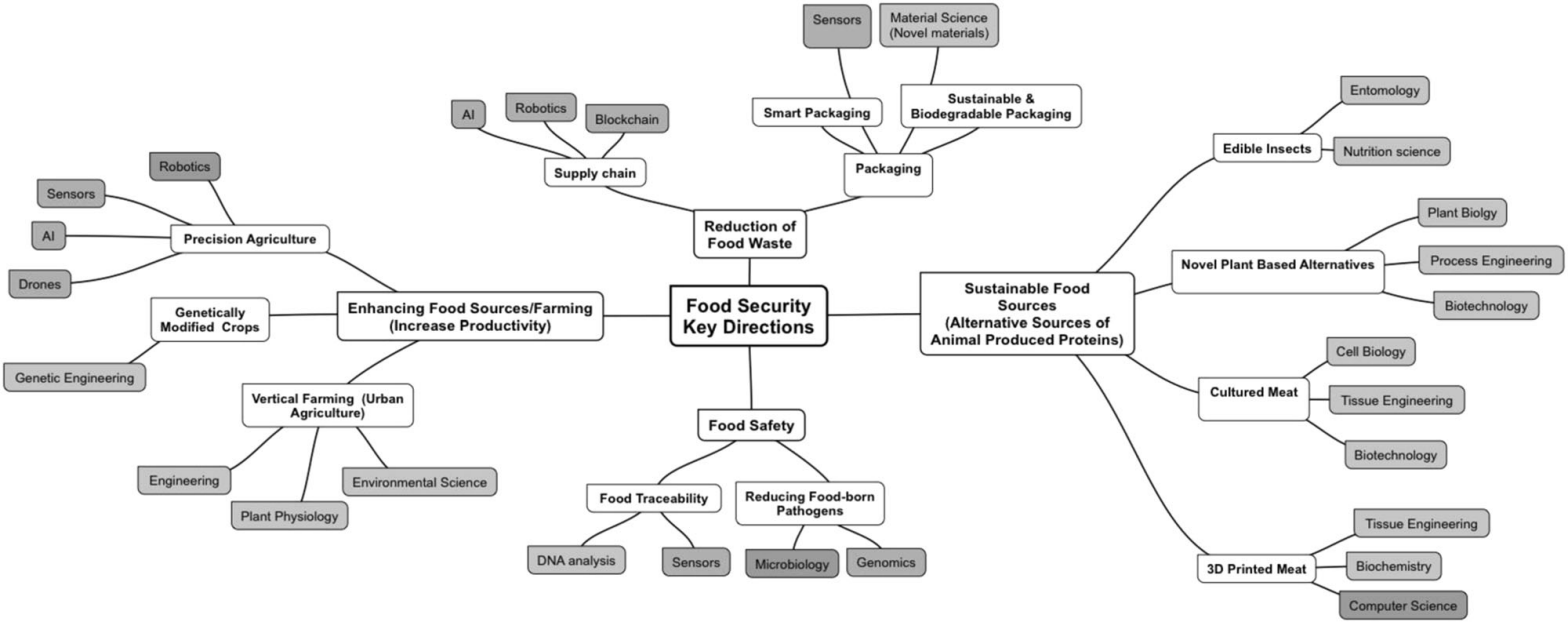
Multidisciplinary approaches to address food security challenge: innovation focal points. Multidisciplinary approaches are required to effectively address the Food Security Challenge which result in innovation technologies and approaches, e.g., advancement in genetic engineering to develop high-yield and disease resistant crops; using drones and big data analytics for precision farming; expanding use of 3D bioprinting to produce non-animal based meat alternatives. Note: *the diagram aims to provide an overview of innovation examples across different scientific areas rather to list all scientific fields involved in the Food Security area*.

Considerable efforts have been put in place to address this challenge ranging from precision agriculture to food innovation (Fig. 1). This involves truly cross disciplinary innovation efforts for example, genetic modifications to increase crops’ resilience^5^, different antibacterial strategies to improve food security^6^, sensors^7^ and big data advances for precision farming^8^, artificial intelligence (AI) and block chain technologies to manage food supply^9^, advancement in material science to develop functional packaging materials^10^, tissue engineering in the development of alternative protein sources^11^ etc.

However, harnessing the potential of such technologies for food security requires the ability to translate them into commercial and scalable applications. In many cases, the first step of this translation is done by startups and/or university spins outs. This is particularly true for the development of alternative protein sources, which has been selected as a topic for this review. Alternative protein sources are used to substitute animal source protein-rich foods and form an integral part of sustainable food systems to meet protein demands that are projected to nearly double by 2050^12^. Two opposing protein transition trends are taking place: low-income populations shift from plant to animal protein sources while high income populations are looking to substitute animal protein sources^13^. Multiple factors are driving this transition including economic growth in lower income countries and having more animal products in their diet^13^, while the raising environmental and health concerns have led to dietary changes in higher income countries^13^. However, it is important to note that these are not conflicting trends, i.e., it is possible to reduce the consumption of meat in high income countries and at the same time increase animal-based protein consumption among the socio-demographic groups for which more protein in their diet would be beneficial^14^.

The current review aims to bring together the latest advancements in the alternative protein sources research and insights into their translation into product applications, i.e., their impact outcomes to address the food security challenge.

## Startups eco-system

### Data source

Startups, which are positioned at the forefront of technological innovation and aim to turn groundbreaking research into product applications and creating new solutions, present a good indicator of the latest technology and research advancements and as such were used as a data source for this review. Due to their agile and lean structure, they can rapidly pivot and iterate to drive technological progress that in many cases are further strengthened by their collaborations and partnerships with academia, established companies and other organisations.

### Eco-system design

**Fig. 2:**
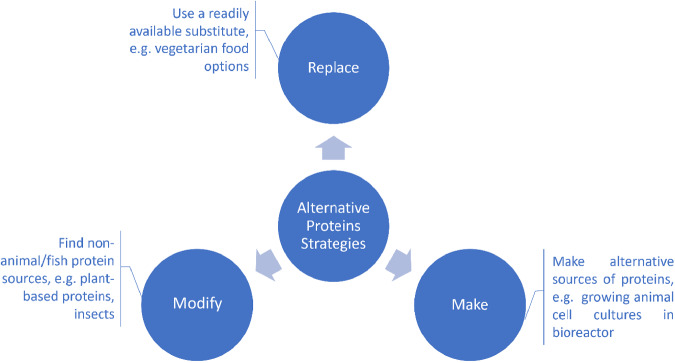
Innovation strategies to develop alternative (non-animal or fish) protein sources for the food industry. A problem-solving innovation strategy approach was used to design the startups eco-system. The problem to solve is to find an alternative to animal/fish-based proteins and when it comes to finding an alternative, there are three main options to consider: (1) using a replacement, (2) modifying existing non-animal/non-fish sources of proteins, and (3) making an alternative source of proteins. (1) Replace: this option involves using a readily available substitute for the target compound, e.g., current vegetarian diet options. (2) Modify: this option looks at modifying existing non-animal/non-fish sources of proteins to substitute the target compound, e.g., insect-based protein. (3) Make: This option comes from a product innovation standpoint, providing the most potential, while holding the biggest challenges. It includes using novel technological processes to make proteins, e.g. 3D bioprinting, cell-cultured products, precision fermentation etc.

Most of reviews dedicated to the development of alternative food sources are structured around the production method^15–18^, e.g. cell-cultured products, precision fermentation, insect proteins etc and/or a specific source of alternative proteins^19–22^. The current eco-system was designed based on an innovation strategy to address a problem – finding an alternative to animal/fish-based proteins. When it comes to finding an alternative, there are three main options to consider: (1) using a replacement, (2) modifying existing non-animal/non-fish sources of proteins, and (3) making an alternative source of proteins (Fig. 2).

1. Replace: this option involves using a readily available substitute for the target compound, e.g., current vegetarian diet options.
2. Modify: this option looks in modifying existing non-animal/non-fish sources of proteins to substitute the target compound, e.g., insect-based protein.
3. Make: This option comes from a product innovation standpoint, providing the most potential, while holding the biggest challenges. It includes using novel technological processes to make proteins, e.g. 3D bioprinting, cell-cultured products, precision fermentation etc.

**Fig. 3:**
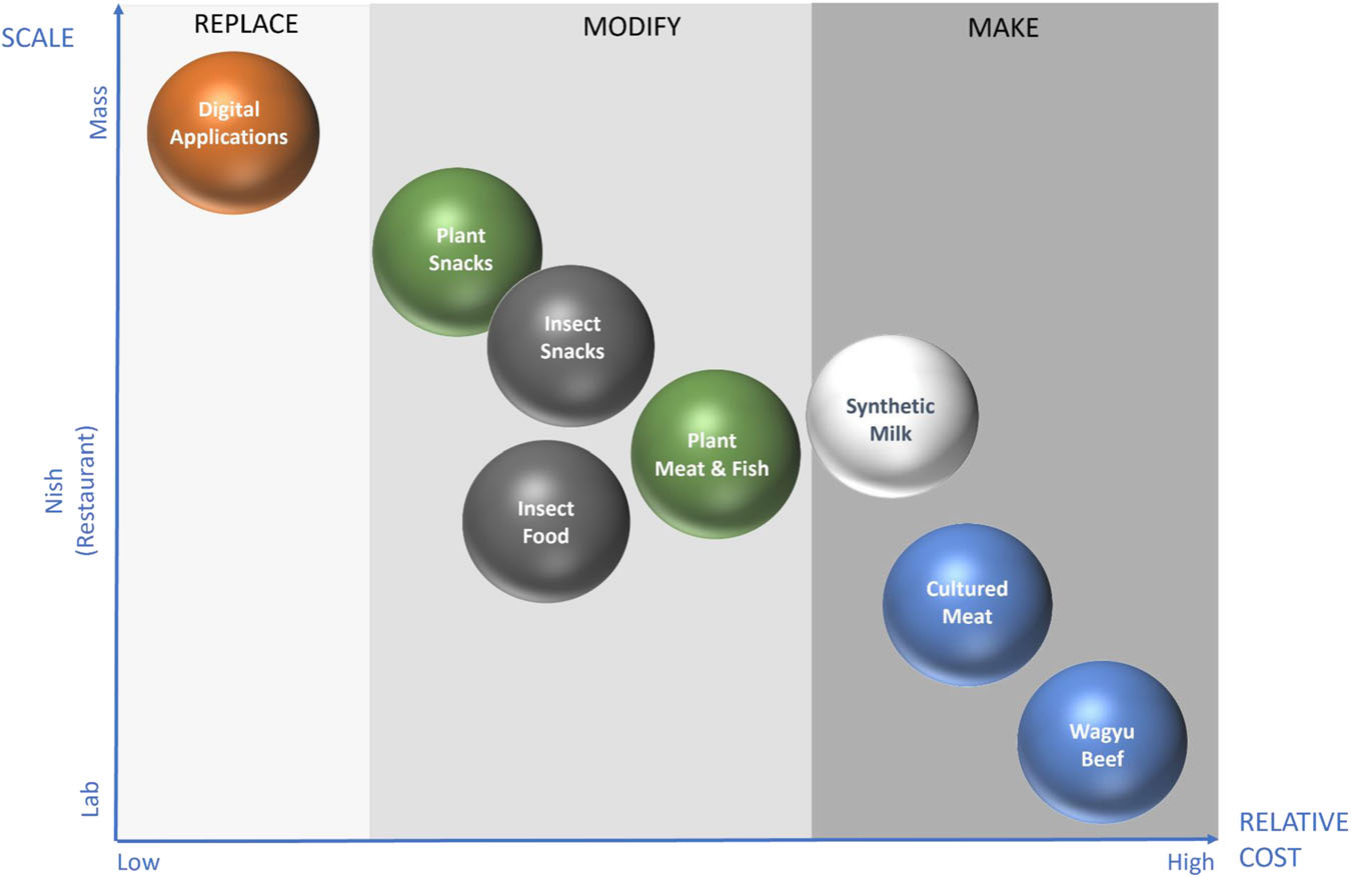
Alternative protein replacement strategies differentiation of market penetration potentials based on cost and scalability. Assuming the same consumer perception of different alternative proteins products, their market penetration would primarily depend on their scalability (ability to move from niche to mass market without compromising quality) and cost (at least parity to the animal/fish-based protein products). These two parameters were used to map market the penetration potential of different alternative protein options using current market examples. *Note:* products examples are intended to illustrate a relative position and presented in a non-scale format.

In terms of the market penetration, scalability and cost were main differentiating parameters between these three options (Fig. 3).

### Eco-system composition

**Fig. 4:**
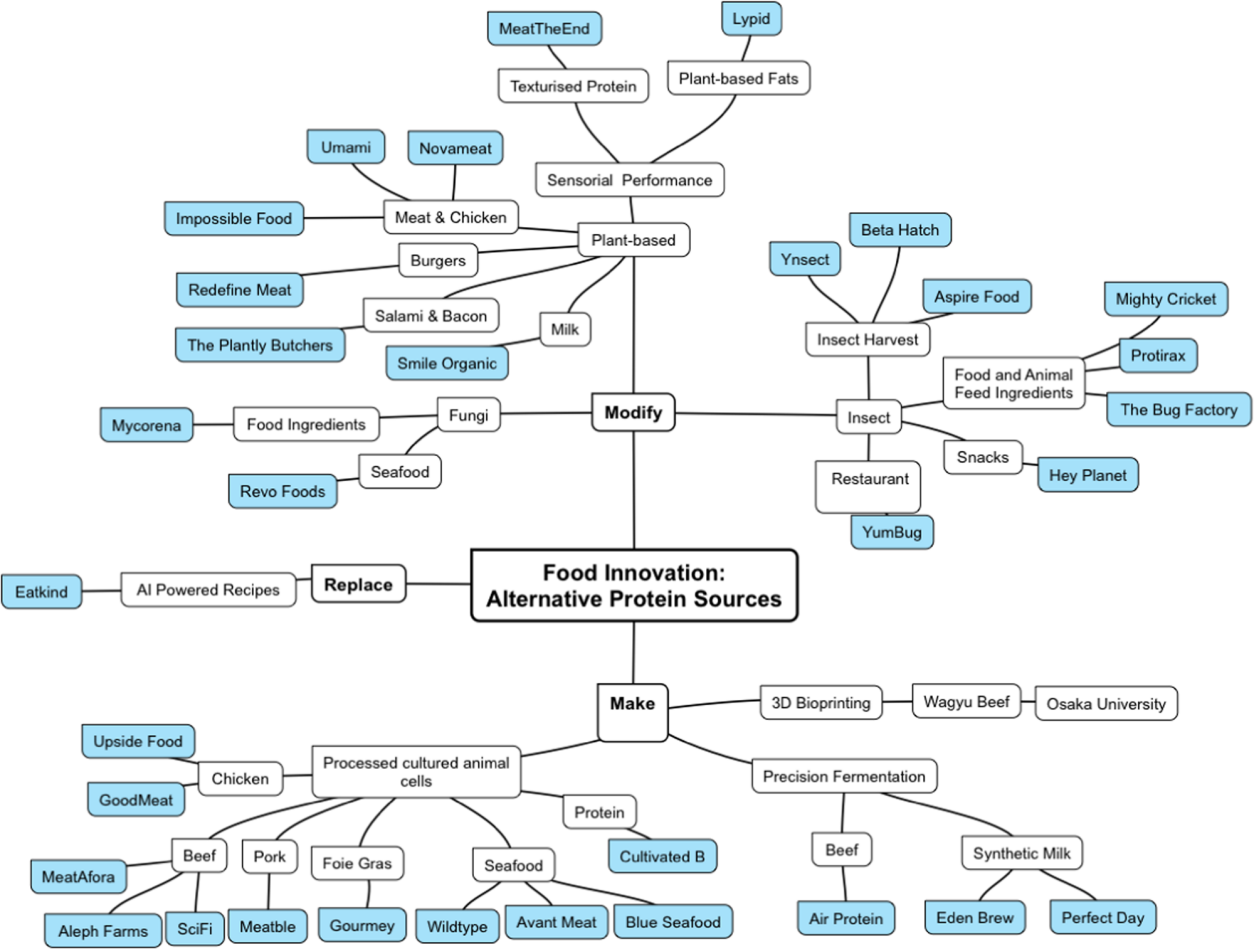
Alternative protein sources startups eco-system. Startups eco-system is designed based on innovation strategy approach used to produce alternative proteins that forms three main pillars of the eco-system: (1) Replace, (2) Modify and (3) Make. Each pillar has 2–3 levels with one-to-two levels specific to each pillar. The specific levels were designed to represent specific features of each pillar, e.g. the first level in the make pillar is the type of manufacturing method/technology approach used to make alternative proteins while in the replacement pillar, it is based on different sources of the alternative proteins (insects, plants, fungi). The second level deals with product’s types, e.g. chicken, beef, salmon, snacks. The last level provides examples of startups selected based on them (i) having an alternative protein product offering; (ii) representing some of the main trends and (iii) being active, i.e. website news posting in 2023 and presented for illustrative purposes only.

For illustrative purposes, 33 startups have been selected to provide a representative sample of the alternative protein startups eco-system (Table 1). The selection process was based on (i) sources of alternative proteins: insects, plant and cells; (ii) technology approach used e.g., extrusion, precision fermentation, 3D bioprinting, cell culture and (iii) the a type of alternative product, e.g. alternative meat, fish, chicken, protein, milk etc. The startups selection was rendered based on them (i) having an alternative protein product offering; (ii) representing some of the main trends and (iii) being active, i.e. website news posting in 2023. (Fig. 4).

**Table 1:** Alternative protein start-ups examples

StartUp	Products	Country	Year Founded	URL
Eatkind	Animal protein replacement menu	India	2021	https://www.eatkind.co/
Ÿnsect	Aquaculture, pets, poultry and swine feed from insects Human food ingredient range derived from insects Plant fertiliser made from mealworm	France	2011	https://www.ynsect.com/
Hey Planet	Insects based protein bars Insects containing snacks Cooking with insects ingredients & recipes	Danmark	2016	https://www.hey-planet.com/
YumBug	Insect-based restaurant & recipes	UK	2018	https://www.yumbug.com/
Beta Hatch	Aquaculture, pets, poultry and swine feed from insects Soil fertiliser made from mealworm	US	2015	https://betahatch.com/
Mighty Cricket	Insect-based protein powder Insect-based protein Oatmeal	US	2018	https://mightycricket.com/
Protirax	Insect ingredients for food and animal feed on an industrial scale	Netherlands	2009	https://protix.eu/
Aspire Food	A wide range of cutting-edge technologies the insect agriculture industry	UK	2018	https://aspirefg.com/
The Bug Factory	Home insects’ breeding kits to produce animal feed	UK	2019	https://bugfactory.co.uk/
Novameat	Plant-based meat: plant-based ingredients mixed with Novameat’s texturising technology	Spain	2018	https://www.novameat.com/
MeatTheEnd	A range of protein premix products to improve transform the texture of alternative meat	Israel	2020	https://www.meattheend.tech
Lypid	Plant-based fats	US	2020	https://www.lypid.co/
Revo Foods	Plant & fungi based seafood 3D salmon fillet	Austria	2020	https://revo-foods.com/
Mycorena	Protein ingredients from fungi	Sweden	2017	https://mycorena.com/
Umiami	Plant-based meat and chicken	France	2022	https://umiami.com/
Redefine Meat	Plant-based burgers	UK	2018	https://www.redefinemeat.com/uk/
Smile Organic	Plant-based alternative to cow’s milk baby food	Canada	2019	https://smileorganic.com/
Impossible Food	Plant-based chicken nuggets, sausage, beef minced and pork	USA	2011	https://impossiblefoods.com/gb-en
The Plantly Butchers	Plant-based salami and bacon	Germany	2020	https://the-plantly-butchers.com/en/
Upside Food	Cultivated chicken meat	USA	2015	https://upsidefoods.com/
GoodMeat/ Eat Just	Cultivated chicken meat	USA	2016	https://www.goodmeat.co/
Aleph Farms	Cultivated steaks	Israel	2017	https://aleph-farms.com/
Meatable	Cultured meat hamburger	Netherlands	2018	https://meatable.com/
Wildtype	Cultured salmon	USA	2016	https://www.wildtypefoods.com/
Avant Meat	Cultivated fish	China	2018	https://www.avantmeats.com/
Bluu Seafood	Cultivated fish	Germany	2020	https://www.bluu.bio/product
Gourmey	Cultivated foie gras	France	2019	https://www.gourmey.com/
SciFi Foods	Cultivated beef	USA	2019	https://scififoods.com/
MeatAfora	Cultivated beef	Israel	2021	https://meatafora.com/
Perfect Day	Dairy ingredient, ProFerm™, based on whey protein	USA	2014	https://perfectday.com/
The Cultivated B	Cultivated protein	Germany	2021	https://www.thecultivatedb.com/
Eden Brew	Animal-free milk	Australia	2021	https://www.edenbrew.com.au/
Air Protein	Air protein meat	USA	2019	https://www.airprotein.com/

These companies have been reviewed across three main vectors: (1) scientific insight behind the technology; (2) product development stage and (3) key challenges.

## Alternative protein sources

### Replace (meat protein replacement)

This option deals with using existing non-animal-derived ingredients to substitute proteins from animals and fish. While from a product innovation standpoint, it may not be considered significantly innovative, the process of considering replacement options utilises the latest developments in computer – digital databases and machine learning. A significant amount of data including food composition, nutritional composition and recipes is publicly available^23–25^. It provides a fruitful ground to utilise machine and deep learning approaches for food design^26^.

Taking this approach, Eatkind Technologies Private Limited (India) has developed an AI-based tool (EatKind^27^) to replace meat, egg and dairy ingredients in a recipe for a plant-based one. The EatKind site turns any recipe into a plant-based one by posting it in the site’s search box.

### Modify (non-meat source of proteins)

The next option is using existing sources of proteins, such as insects and plants, as non-animal or/and fish proteins sources.

#### Insect based

Entomophagy, the consumption of insects as food, has been a common practice in many cultures for centuries^28^. Insects have great potential as a sustainable animal protein source due to their low impact on resources, e.g. emitting less greenhouse gases, requiring less water and space^29^.

The data from various comparative analyses^30–32^ of the protein and other nutrients content in edible insects and animal-derived meat have demonstrated that both edible insects and animal-derived meat have varied nutritional content with more profound variations in edible insects^30^. The latter was considered to be due to the diversity of individual species^32^. Edible insects have a higher protein content than animal meat^31^, ranging from 23.1 g to 35.2 g per 100 g among edible insect species and 19.2 g to 21.5 per 100 g in different types of meat^31^. In regard to the nutrition value, it seems that it is not possible to explicitly state that edible insects would have a higher nutritional value than animal-derived meat, because of the differences in the content of individual nutrients in edible insects and animal-derived meat^30,31^. In addition to this, this analysis would be impacted by using different nutrient profiling models^30^.

An estimated 2 billion people^33^ across Africa, Asia, Central and South America, and Australia consume insects and there is an increased interest in Western countries in insects as a potential source of food. The Edible Insects Market size is estimated at USD 3.20 billion in 2023, and is expected to reach USD 7.60 billion by 2028, growing at a compound annual growth rate of 18.89% in 2023-2028^34^. Two main factors contribute to this trend: (1) the growing acceptance of insect-based food in Western societies^35^ and (2) their lower environmental impact to address the food security challenge. This rapid growth is supported by increasing investments. While the edible insects’ market is highly fragmented^34^, it has attracted more than USD 1.3 billion in funding to date with more than half of it in the past couple of years^36^. Increasing investment in the startups’ research and development (R&D) also comes from partnerships with existing companies. For example, Protirax announced a strategic partnership with Tyson Foods, one of the world’s largest food companies^37^, Ÿnsect launched a dog feed brand in the US in collaboration with Pure Ultra Simple LLS, a dog food start-up (US)^38^.

To help scale up the edible insect-based business globally, many government organisations are developing programmes and initiatives including a collaboration between the Australian Centre for International Agricultural Research (ACIAR), AgriFutures Australia and the International Centre for Insect Physiology and Ecology (ICIPE) resulting in the creation of the Emerging Insect Technology Hub (EIT-Hub) that aims to bring together industry stakeholders, scientists and investors to discuss issues linked to emerging insect technologies around insects as food, animal feed and fertiliser^39^. The other example is the ‘Insectrial Revolution’ project which received USD 7.5 million from the UK government’s Industrial Strategy Challenge Fund (ISCF). This project focusses on the construction of the country’s first large-scale industrial insect farm run on food waste. It is being led by led by the insect-farming company Entocycle (UK) and brings together a consortium of 15 partners providing their diverse expertise and ranging from academic partners and multinational companies, e.g. insects technology expertise (BetaBugs, Better Origin, Fera); science (Durham University, University of Stirling, University of Warwick, Scottish Aquaculture Innovation Centre); waste management (not-for-profit environmental organisation Zero Waste Scotland) and a multinational company (Tesco)^40^.

Following the development of relevant regulatory frameworks and legislations covering edible insects, the companies (examples are given in Table 1) were able to place their product in the market, making them available to consumers. For example, in the United States, edible insects and insect-based food products must comply with the Federal Food, Drug, and Cosmetic Act (FD&C Act)^41^, in the European Union all insect-based products (whole insects, their parts or extracts) meant for human consumption have fallen under the novel food regulation EU 2015/2283^41^ and in the United Kingdom the Food Standards Agency (FSA) is now requiring insect companies to submit dossiers of evidence of safety^42^.

Insect-based food startups’ activities range from harvesting the insects to producing food products (examples are given in Table 1). These companies’ development is a result of a multi-disciplinary effort encompassing entomology (various rearing techniques), together with food and nutritional science (product formulation and processing methods). For example, Ÿnsect^43^, a French startup, has the largest vertical farm in the world and its recent innovation is a genotyping chip Axiom® YNS_Mol1 for insect breeding aid selection of larvae lines to produce insect-based proteins. This chip has been made available for companies and the scientific community. Big data genome analytics, RNAi and CRISPR are used by Beta^44^ to customise their insects^44^. Other startups are working to integrate insect powders into the Western world diet by developing products palatable to the Western taste preferences, e.g., protein bars, chocolates, and beetle-based meats by Hey Planet^45^ and/or YumBug^46^ opening an insect food-based restaurant.

The farming of insects for feed and the production of insect-based foods are relatively recent and bring both benefits and challenges. As with other foods, potential food hazards^41,42^of insects-based food could include biological agents (bacterial, viral, fungal, parasitic), chemical contaminants (pesticides, toxic metals, flame retardants), potential allergic reaction, in particular in individuals with crustaceans’ allergies to allergen cross-reactivity.

The high nutritional content and the low carbon, water and ecological footprints associated with insect production, as compared to those of other livestock species, make them an attractive protein replacement option for a healthy diet both for animals and humans. However, further studies and monitoring will be required to determine their quality and safety^41^. From the companies’ perspectives more efforts will be required to increase broader consumers’ acceptability of insect-based food and address the current key barriers dealing with neophobia and repulsion (the yuck factor) with insect food^47^. The main focus areas to address these barriers include information dissemination about benefits and how to incorporate the insect-based food and improving sensorial experience by developing appealing products.

#### Plant based

Humans have consumed plant-based protein food since ancient times. Records of using soybean in ancient kitchens to produce soybean milk as well as preparing tofu from coagulated soybean milk go back to the Han Dynasty in China^48^. Advancements in processing technology^19^, in particular, sheer cell, extrusion, structuring processes aiming to develop a fibrous structure, the development textured vegetable proteins, as well as ability to address environmental and food security challenges, have resulted in a significant increase in the consumption of plant-based foods. These methods also enable better mimicking of animal source foods by plant-based meat analogues/alternatives (PBMA) and plant-based dairy analogues/alternatives (PBDA). Currently, there are more than one thousand companies operating in this space with 40% of them focussing on PBMAs and PBDAs food production^49^. Over the last decade there has been a rapid rise in the number of PBMA and PBDA startups with 80% of the current companies in this sector being established during this period^49^. The same trend is also observed across different players ranging from academia research to large food companies. It is becoming a subject of numerous articles and review papers^19^ that look at different aspects of producing PBMA and PBDA food including technological developments, life cycle impact assessments to evaluate the sustainability of plant-based meat products, the health benefits, consumers’ perceptions etc. Large food companies recognise the importance of alternative proteins and are increasing their investment as well launching plant-based version of their popular products including dairy-free Philadelphia cream cheese by Kraft Heinz and Mondelez International, Kellogg’s plant-based chicken waffle Eggo sandwich and Burger King’s Impossible burgers^50^. The presence of large food companies has a profound effect on the plant-based alternative market and is driving its consolidation, e.g. The Kellogg Company, Maple Leaf Foods and Conagra Brands taking nearly 70% of the plant-based meat sales in the US with the Kellogg Company accounting for almost 50% of the total sales^51^. Plant-based meat, seafood, eggs, and dairy companies attracted USD 1.2 billion investment in 2022 and the number of unique investors in plant-based companies grew by 17 percent and reached more than 1500 investors^50^. Similarly, to the edible insect category, the plant-based meat alternatives receive significant support from the public sector, e.g. the German government’s promise to invest USD 41 million in plant-based foods and alternative proteins^52^. Denmark, Sweden, and Switzerland committed to invest more than USD 150 million into plant-based protein R&D^50^.

The newest versions of PBMA have similar textures, comparable smells, and appearance to help mimic animal meat. The Spanish startup, Novameat^53^ uses 3D bioprinting to create fibres and microfibres that unlock the texture of meat and provide versatility to develop a range of PBMA products. To solve the texture challenge, the Israeli startup MeatTheEnd^54^ has developed a proprietary technique to incorporate a unique pre-treatment step prior to extrusion to produce texturized protein ingredients. In combination with extrusion technology that is used in mass-scale production, this method results in a cost-effective solution for PBMA companies that seeks to improve the texture of their products. Lypid (US) is looking to address the sensorial and nutritional challenges of PBMA by providing plant-based fats. It uses encapsulation technique to produce emulsion of plant oils (‘alternative fat’) that behaves like animal fat^55^.

Another source of alternative proteins are fungi that includes microorganisms such as yeasts and moulds with mushrooms being the most familiar form. A number of startup companies use filamentous fungi as a source of microproteins. For example, Revo Foods (Austria) has developed a proprietary extrusion process and fibrous protein matrix from filamentous fungi to produce 3D-printed salmon-like fillet on a commercial scale^56^ while Mycorena (Sweden) is using a liquid fermentation process to produce fungi-based protein food ingredient and also fungi-stabilised fat that can be used to improve the sensorial performance of PBMA products^57^.

A cross-sectional analysis^58^ of more than 200 products in each product category, PBM and meat, demonstrated that PBM products had significantly lower protein content than meat products, for example, mean protein content per 100 g in meat sausages was 15 g and 12.1 g in PBM sausages; in meat burgers 19.9 g in PBM burgers and 23.3 g in plain chicken and 18.7 g in plant-based chicken. However, according to the UK’s Nutritional Profiling Model, more PBM products were classified as healthier than meat products, i.e. 14% of PBM and 40% of meat products were classified as ‘less healthy’ (*p* < 0.001)^58^. Future studies are needed to better understand how the presence and absence of metabolites and nutrients in plant-based meat alternatives and meat impacts short- and long-term consumer health.

Technological advances have enabled the field to address a range of critical issues; however, there are still a number of challenges including scalability and cost that remain. The main challenges around PBMAs include allergy concerns associated with soy and wheat; requiring additional flavouring ingredients to achieve the meaty flavour; ability to incorporate fat into the product and potentially a higher risk than meat products of microbial growth due to high-moisture environments with a neutral pH^59^. In terms of PBDAs, they have some performance issues dealing with stability and removal of off flavours, e.g. a beany flavour, bitter taste, and astringency. In addition to the above, there is limited scientific data related to the safety of PBMAs and PBDAs.

### Make (made/produced protein sources: lab grown meat)

Creating a meat substitute is perhaps the most challenging option as it involves the production of meat in vitro. The foresight of growing meat outside an animal environment in 1931 when in his speech ‘Fifty Years Hence’, Winston Churchill said ‘We shall escape the absurdity of growing a whole chicken in order to eat the breast or wing, by growing these parts separately under a suitable medium.’^60^ The latest progress in cross disciplinary efforts including tissue engineering, stem cell biology, bioprocess engineering has made this feat possible. There are currently more than 150 companies^20^ working to produce lab grown meat, which typically follow one of the following technologies routes: (1) processing cell cultures grown in bioreactors; (2) 3D-bioprinting or (3) precision fermentation (Fig. 4).

#### Processed cell cultures

Harrison’s pioneering work^20^ in 1907 led to the development of the cell culture techniques. The progress made in more than 100 years of research in this field resulted into it becoming a widely used research tool^21^ as well as a wide range of biotechnology applications including the tissue engineering, regeneration medicine, cells for vaccine and cell therapy. ‘Cultured meat’ presents one of the latest developments in the field of cell culture. The approach relies on growing and expanding animal stem cells inside a bioreactor and then using them to produce cultivated meat. In the early 2000s the first world’s research institute dedicated to cultured animal products was formed and it took just over 20 years from a cultured meat concept to a cultured meat product on the market.

In recent years there has been a considerable rise of cultured meat startups with 156 companies operating in this space by in 2022 and USD 2.8 billion all-time investment in cultivated meat and seafood companies^12^. Their products range from common meat such as chicken (Upside Food^61^ (US), GoodMeat/Eat Just^62^ (US)), beef (Aleph Farms^63^ (Israel)), pork (Meatable^64^ (Netherlands)) to seafood (Wildtype^65^ (US), Avant Meat^66^ (China) and high-end food including foie gras (Gourmey^67^(France)).

In the 2016-2022 period, the cultivated meat and seafood companies attracted USD 2.78 billion^12^. The funding streams include unique investors reaching 679 investors^12^, strategic partnerships between cultivated meat companies and major food companies with at least 35 major partnerships^12^ including established companies like Nestlé, Merck KGaA, Mitsubishi, JBS, Kerry, and CP Kelco^12^ and public funding. For example, Singapore’s government launched a number of programmes to support alternative protein startups and accelerate innovation including building the world’s first hybrid innovation centre dedicated to cultivated and plant-based meat products^68^. The development and manufacturing of alternative proteins, including cultivated meat, is a part of the UK Government £2 billion National Vision for Engineering Biology plan^69^.

Cultured meat is still not widely available, and a number of hurdles^22^ need to be overcome before it will become a product that is readily available to the masses. These challenges include the lack of regulatory guidelines: regulatory approvals are required to sell a product and many countries do not have established protocols for certifying cultured meat. The startups are navigating the current regulatory landscape, e.g., Singapore was the first country that approved cultivated chicken for public sale in 2020^70^, since then two startups, Upside Foods and Eat Just, received FDA’s approval^61,62^for their lab-grown chicken. Aleph Farms is seeking regulatory approval to sell its beefsteak in the UK and Switzerland^63^. To help facilitate the development of regulatory protocols, more understanding would be required to assess potential risks of microbial contamination, genetically engineered starting materials etc to address any food safety concerns. The other challenges include sensorial and visual acceptance and cost. For example, to develop a satisfying prototype version that could match the delicate flavour and creamy texture of foie gras, Gourmey tested 600 to 650 different compound interactions. Its product will be marketed as a “poultry delicacy as it cannot be called foie gras” in France. Wildtype is another startup that successfully managed the sensorial challenge and created sushi-grade salmon by cultivating cells extracted from salmon eggs and it has been reported that the resulting tastes like conventional sushi-grade salmon^65^. However, it comes at a cost - the 127 g (4.5 ounce) portion cost about USD 150 in food costs alone (USD 533 per 450 g (1 pound)).

Limited information is available about the protein content in cultured meat^22^ and according to morphological observations, there are indications that the current cultured meat with most of the cytoskeletal proteins is in the same range as traditional meat^71^.

The cost represents another challenge of cultivated meat, which will decrease as technology improves to enable scale up. There is already evidence on making scalable beef cell lines using CRISPR by SciFi Foods^72^, a US based cultivated meat startup, which aims to eventually reach USD $1 per burger at a commercial scale. SCiFi noted that their technology allowed the cost reduction of cultivated meat by more than 1000 times compared to current production costs and less than USD 10 cost for its blended burger, part plant-based and part cultivated meat, that is 33,000 times less than the first cultivated burger developed by Mark Post and Peter Verstrate less than a decade ago that had a production cost of USD 330,000^73^.

#### 3D bioprinting

Additive manufacturing, the process of joining materials to make objects from computer-aided design model data, such as 3D printing, have opened tremendous opportunities in a broad spectrum of applications in several industry sectors. The integration of 3D printing into tissue engineering provides opportunities for many innovation solutions including regeneration medicine, in-vitro models, pharmaceutical and food industries and healthcare challenges and heralds’ new frontiers in medicine, pharmaceutical, and food industries^74^.

Scientists from Osaka University used this method to print Wagyu beef^75^ that resembles the real pieces of meat and reproduces its complex structure formed by muscle fibres, fat, and blood vessels. The fibres fabricated from stem cells using bioprinting were then arranged in 3D to reproduce the structure of the real Wagyu meat and sliced perpendicularly, like the traditional Japanese candy Kintaro-ame. Tendon-gel-integrated bioprinting used for the fibre cells’ fabrication could expand a culture meat toolbox and provide a valuable approach for constructing engineered steak-like meat.

#### Precision fermentation

The advancements in genome-based technologies enabled a transformation of the traditional fermentation process and development of precision fermentation that uses microbial hosts as ‘cell factories’ for producing specific functional ingredients^18^. This process has been used to produce animal-based proteins and can save water and land compared to traditional livestock farming with the added benefit of zero methane gas emission. For example, startups Perfect Day (US)^76^ and Eden Brew (Australia)^77^ use this approach to produce the same proteins found in cow milk and create synthetic milk that have similar taste, look, and feel to dairy milk. While the technology has significant potential to address environmental challenges, there is an on-going discussion of its downsides, for example, in the case of synthetic milk, its potential impact on to the dairy industry and conventional agriculture and the prospect of pushing out low-tech or small-scale dairy farms. However, to do so, the industry must grow exponentially and build new manufacturing infrastructure (e.g., fermentation tanks, bioreactors) that would require a considerable amount of investment.

Another startup that uses precision fermentation is Air Protein (US)^78^. It uses microbes to transform carbon dioxide from air into meat that originates from the 1970s space programme where NASA scientists explored a way to feed astronauts on long space journeys by transforming elements in the air that the astronauts breathed into proteins^79^. The end fermentation product is a versatile protein-rich flour, which has a similar amino acid profile as meat protein and can be turned into any food using a combination of pressure, temperature, and other technologies. The manufacturing process has climate-saving potential: it is carbon-negative and compared to beef, uses 1.5 million times less land and reduces water usage 15,000 times. Like with other cultured meat challenges, the most crucial aspect is making the process cost competitive. Published techno-economic analysis indicated a production range in a hypothetical commercial-scale facility ranging from USD 17 to USD 65 per kilogram where the largest cost drivers include culture media, bioreactors, and labour^12^.

## Conclusion

New technologies and discoveries continue to fuel the field of alternative protein sources. Alternative protein sources used to fabricate meat, fish and diary food products can help to address food security challenges and mitigate environmental issues, by significantly reducing emissions and requiring far less land. For example, based on a life cycle assessment, plant-based meat can cut emissions by 90 percent, and use 99 percent less land and water compared to conventional meat^80^.

**Fig. 5:**
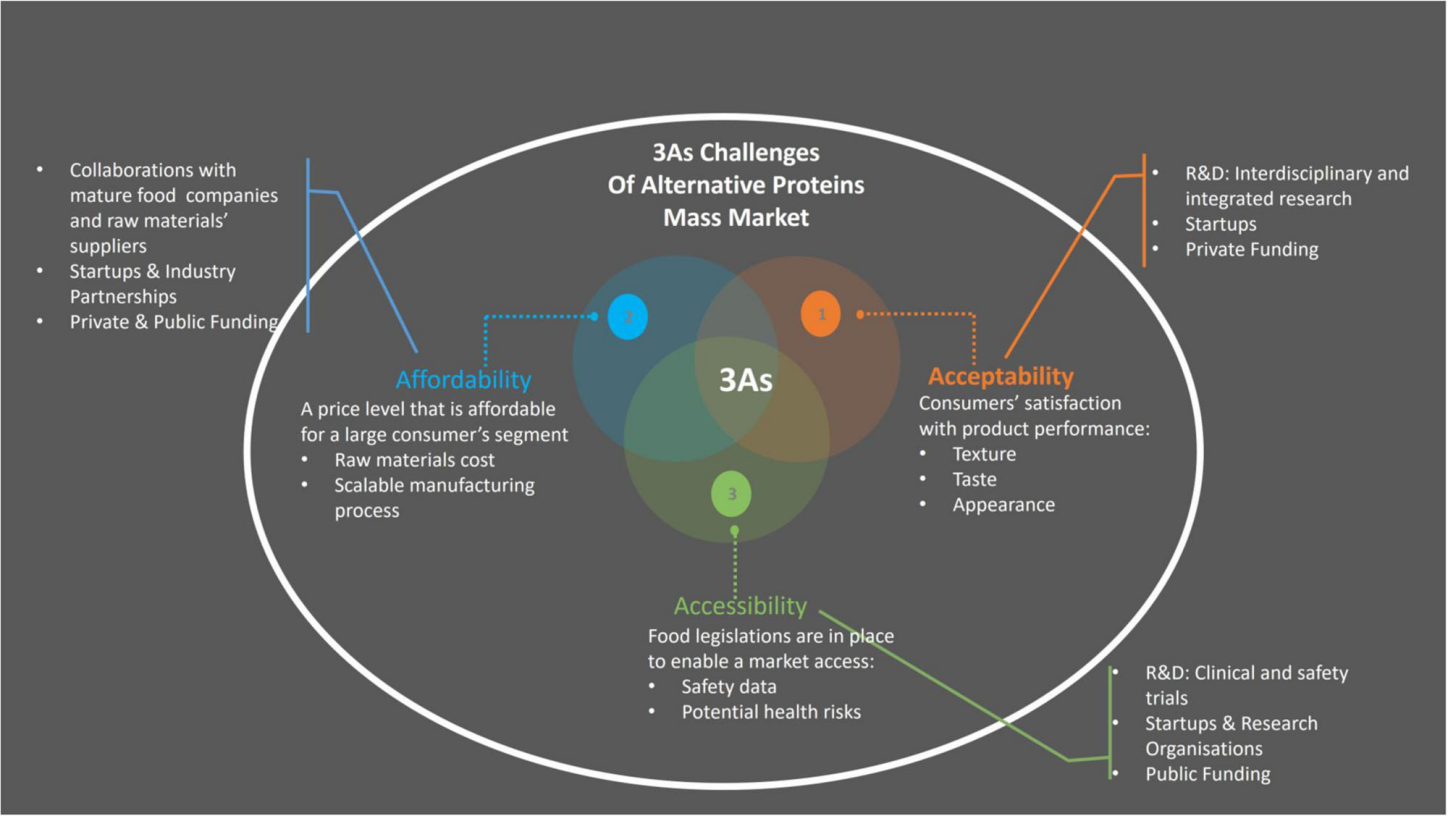
Alternative proteins food: mass market challenges and approaches to tackle them. The main challenges for alternative proteins food to become a commodity could be divided into three groups: (1) Affordability, (2) Acceptability and (3) Accessibility that form 3As framework. Affordability challenge is driven by high cost associated with a complex technological process and its scalability as well as raw materials cost. Acceptability challenge deals with consumer acceptance of alternative protein proteins products and their ability to mimic traditional food in terms of texture, taste, and appearance. Accessibility challenge is ability to place these products on the market and rely on a regulatory framework that is in a development stage and requires more research to demonstrate their long-term safety profile and potential health risks. Multi-dimensional efforts are being put in place to address these challenges that include interdisciplinary and integrated research, new partnerships and global alliances and continuous investment from both private and public sources. Specific examples of these activities are presented for each of the 3As challenges.

The current alternative proteins sources include insect-, fungi- and plant-based proteins and cell-based proteins grown from animal cells in the form of cultivated meat and fish. Producing food from alternative proteins has made a big leap forward in the past decade with plant-, fungi- and insect-based food available in grocery shops and food made from meat substitutes being served in restaurants. However, there are still a number of challenges to overcome before these products will become a commodity accessible to everyone. The main challenges (Fig. 5) include consumer acceptance driven by their ability to mimic traditional food in terms of texture, taste and appearance; affordability due to high cost associated with a complex technological process and its scalability as well as raw materials cost and a development of regulatory framework to enable market accessibility that requires more research to demonstrate their long-term safety profile and potential health risks.

Interdisciplinary and integrated research, new partnerships and global alliances and continuous investment from both private and public sources^81^ will enable these issues to be addressed. The Importance of alternative protein sources to achieve national economic growth, sustainability and food production objectives have been recognised by both government and non-government organisations including forming cross-stakeholders’ partnerships, e.g. the U.S.-based Alliance for Meat, Poultry, and Seafood Innovation, the APAC Society for Cellular Agriculture, and Cellular Agriculture Europe launched a new global alliance to collaborate on regulatory work, consumer research, and nomenclature^12^.

The above creates fertile ground for startups in this sector to continue evolving and to deliver breakthrough innovation by bringing fresh perspectives, creativity, a willingness to take risks, targeting niche issues (in particularly, sensorial perception barriers) and their ability to bridge between the latest advancements in academic research and the go-to-market resources of large companies.
